# Association of TILs and HER2-low status with pathological response in triple-negative breast cancer

**DOI:** 10.1097/MD.0000000000043971

**Published:** 2025-08-15

**Authors:** İlknur Deliktaş Onur, Didem Kurukafa, Yeşim Ağyol, Hatice Gülgün Firat, Elif Sertesen Çamöz, Alper Türkel, Berkan Karabuğa, Sibel Yenidünya, İbrahim Vedat Bayoğlu, Öztürk Ateş

**Affiliations:** a Department of Medical Oncology, University of Health Sciences, Dr. Abdurrahman Yurtaslan, Ankara Oncology Education and Research Hospital, Ankara, Turkey; b Department of Pathology, University of Health Sciences, Dr. Abdurrahman Yurtaslan, Ankara Oncology Education and Research Hospital, Ankara, Turkey; c Department of Medical Oncology, University of Marmara, Istanbul, Turkey; d Department of Internal Medicine, University of Health Sciences, Dr. Abdurrahman Yurtaslan, Ankara Oncology Education and Research Hospital, Ankara, Turkey.

**Keywords:** HER2 low breast cancer, HER2 zero breast cancer, pathological complete response, TILs, triple-negative breast cancer, tumor infiltrating lymphocytes

## Abstract

Triple-negative breast cancer (TNBC) is the most aggressive breast cancer subtype with the lowest treatment success rate. The aim of this study is to determine the relationship between tumor-infiltrating lymphocytes (TILs) score and HER2 score in TNBC patients receiving neoadjuvant chemotherapy and its effectiveness in predicting treatment response. One hundred two patients diagnosed with TNBC, who received neoadjuvant chemotherapy and underwent surgery were included in the study. The TILs score in the tru-cut biopsies of the patients was evaluated, and pathological response rates from mastectomy materials were also examined. The pathological complete response (pCR) was observed after neoadjuvant chemotherapy in 43 (43.2%) patients, while no pCR in 58 (56.8%) patients. Chi-square analysis revealed a significant relationship between the TILs score (p: 0.001), clinical T stage (*P* = .019), and pCR response. In multivariate logistic regression analysis, a significant relationship was found only between TILs and pCR (OR = 13.3, 95% CI = 2.23–81.23, *P* = .00). No significant relationship was found between pCR and HER2 score (*P* = .23), Ki-67 (*P* = .52), grade (*P* = .87) and menopausal status (*P* = .20). No significant relationship was found in the correlation analysis between TILs and HER2 score (*P* = .26, CC = 0.14). TILs score in TNBC is related to both the pCR and disease prognosis. We believe that larger-scale studies are needed to identify more factors that may predict neoadjuvant treatment response in TNBC patients. Additionally, we suggest that the relationship between HER2 score and TILs score in TNBC patients should be examined in larger studies.

## 1. Introduction

Triple-negative breast cancer (TNBC) accounts for approximately 15% of all breast cancers and is more common at younger ages than other subtypes.^[1,2]^ Approximately 16% of all TNBC patients are under the age of 40.^[3]^ It is the most aggressive subtype of breast cancer.^[4,5]^ Therefore, studies are ongoing to improve treatment options. As a result of studies conducted in recent years in patient with non-metastatic TNBC, the standard treatment has shifted from adjuvant to neoadjuvant therapy. Recent developments in the treatment of TNBC have shown that the addition of pembrolizumab to standard chemotherapy in the neoadjuvant period increases pathological complete response (pCR) rates.^[6]^

Tumor-infiltrating lymphocytes (TILs) represent the sum of mononuclear inflammatory cells over the total intratumoral stromal area. This parameter is calculated as the percentage of stromal lymphocytes visually assessed on hematoxylin and eosin (H&E) sections. The degree of lymphocytic infiltration assessed by simple evaluation of H&E-stained tumor sections has predictive and prognostic value in TNBC. A high TILs score is thought to increase immunogenicity in solid tumors and may predict response to immunotherapy.^[7]^ In a meta-analysis that included 37 studies, TNBC patients with high TILs scores had a higher rate of pCR with neoadjuvant therapy compared to those with low TILs levels. This is also associated with PFS and overall survival time.^[8]^

In recent years, the definition of HER2 score has been investigated for TNBC. Patients with HER2 1 + and 2 + on immunohistochemical (IHC) examination and negative FISH results were classified as HER2-low. According to IHC, patients with HER2 0 were classified as HER2-zero. Several studies have compared treatment response and survival in HER2-low and HER2-zero subgroups of patients with TNBC. In recent studies, the relationship between survival difference and treatment response has not been clearly determined, and discussions continue.^[9]^

The aim of this study was to evaluate the relationship between TIL score and HER2 score and its relationship with pCR in patients with TNBC receiving neoadjuvant chemotherapy. Although there are studies in the literature investigating the effects of TILs and HER2 score on pathological response in TNBC, no study has evaluated the relationship between TILs and HER2 score.

## 2. Materials and methods

### 2.1. Study population

One hundred two patients diagnosed with TNBC, aged 18 years and older, who received neoadjuvant chemotherapy and underwent surgery, were included in the study. Patients for whom sufficient data could not be obtained from their files and those for whom tru-cut biopsy preparations were not available in the pathology department archives were excluded from the study.

### 2.2. Data collection

The study was conducted across 2 centers. Patient files were retrospectively examined. Age, sex, date of diagnosis, menopausal status, family history of breast cancer, clinical stage at diagnosis, pathological features of the tumor in tru-cut biopsy, chemotherapy regimen received, response to neoadjuvant treatment, and the date of progression, if any, were recorded. Approval was obtained from the ethics committee of our hospital prior to the start of the study. The ethics committee approval code was 2024-04/52.

### 2.3. Variables measurement and definition

**Figure 1.**
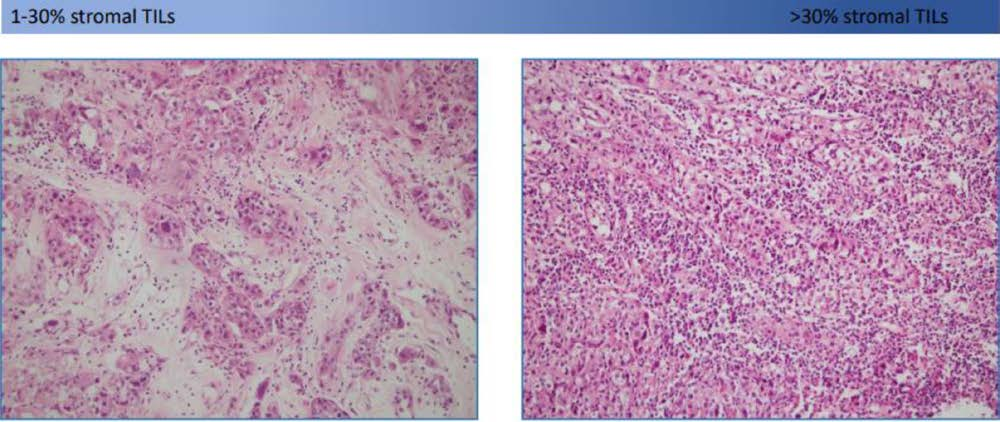
Light microscope images of 1% to 30% stromal tumor infiltrating lymphocytes (TILs) and > 30% stromal TILs.

In this study, the 2014 recommendations of the International TILs Working Group were considered.^[10]^ Evaluation of the tru-cut materials sampled before neoadjuvant treatment was undertaken. All tumor-containing H&E-stained sections were examined under a light microscope by 2 pathologists. The percentage of mononuclear inflammatory outcomes (lymphocytes and plasma distribution) in the stroma between the tumor distributions was determined. Scoring was based on the average TIL percentage, not the hotspot. TILs scores were categorized as 0% negative, 1% to 30% low, and > 30% high. HER2 overexpression was evaluated by immunohistochemistry (IHC). FISH was performed only in patients with 2 + IHC results. The pathological response rates from mastectomy materials were also examined by the same pathologists, and the patients were divided into 2 groups: those with and without a pathological complete response. TILs score light microscope images are shown in Figure 1.

### 2.4. Statistical analysis

All analyses were performed using SPSS version 23.0. In the descriptive statistics of the study, continuous variables were expressed as mean (standard deviation) and median (range); categorical variables were presented as frequencies (percentages). Chi-square or Fisher exact tests were used to compare the categorical variables of the 2 independent groups. The relationship between variables and pathological response was evaluated using the chi-squared test. Statistical significance was set at *P* < .05. Univariate and multivariate binary logistic regression analyses were applied to significant variables. The Spearman test was used for correlation analysis of variables that were not normally distributed. Overall survival (OS) was defined as the time from diagnosis to death or the last visit for patients who were still alive. Progression-free survival (PFS) was defined as the time from diagnosis to relapse. Survival data were updated in April 2024. The association between the variables and PFS was evaluated using univariate analysis. Variables with *P* < .05 in univariate analysis were considered significant and were evaluated in multivariate analysis. In multivariate analysis, Cox regression was used to check the hazard ratio of significant variables.

## 3. Results

### 3.1. pCR and clinicopathologic correlates

A total of 102 patients were included in this study. The median patient age was 52 years. The demographic characteristics of patients are shown in Table 1. pCR was observed after neoadjuvant chemotherapy in 43 patients (43.2%), and no pCR was observed in 58 patients (56.8%). A significant relationship was found between TILs score and pCR response (*P* = .001). A significant relationship was observed between clinical T (cT) stage and pCR (*P* = .019). No significant association was found between the HER2 score (*P* = .23), Ki-67 (*P* = .52), grade (*P* = .87), menopausal status (*P* = .20), and pCR. Multivariate analysis showed a significant association between TILs score (OR = 13.3, 95% confidence interval (CI) = 2.23–81.23, *P* = .00), and pCR. No significant association was found cT stage (OR = 0.4, 95% CI = 0.10–2.11, *P* = .32) and pCR. These relationships are shown in Table 2.

**Table 1:** Demographic characteristics of patients.

	N (%):102 (100)
Age, median, IQR	52 (50–55)
Menopausal status	
Pre-perimenopause	56 (45.1)
Postmenopause	46 (54.9)
Familial breast cancer	
Yes	81 (82.7)
No	21 (17.3)
cT stage	
T1	11 (10.8)
T2	76 (74.5)
T3	11 (10.8)
T4	4 (3.9)
Clinical axillary lymph node involvement	
No	25 (24.5)
Yes	77 (75.5)
Clinical stage	
II	37 (36.3)
III	65 (63.7)
TILs score, median, IQR	5 (9–39)
TILs score	
0	16 (15.7)
1–30	62 (60.8)
>30	24 (23.5)
HER2 status	
0	84 (82.4)
1	11 (10.8)
2	7 (6.9)
Ki-67, median, IQR	70 (30–50)
Grade	
Grades 1 and 2	20 (19.8)
Grade 3	81 (80.2)
Neoadjuvant treatment	
AC + PT	31 (30.3)
AC + T	71 (69.6)
pCR	
Yes	43 (42.2)
No	59 (57.8)
Adjuvant treatment	
No	44 (43.2)
Capecitabine	58 (56.8)

AC = adriamycin + cyclophosphamide, HER2 = human epidermal growth factor 2, IQR = inter quantile range, pCR = pathological complete response, PT = platin + taxane, T = taxane, TILs = tumor-infiltrating lymphocytes.

**Table 2:** Relationship between variables and pCR.

	pCR (n, %)	Non pCR (n, %)	Univariate analysis	Multivariate analysis	
			*P*	OR (95 % CI)	*P*
TILs score			.001	13.3 (2.23–81.23)	.00
0	7 (43.7)	9 (56.3)			
1–30	14 (22.9)	47 (77.1)			
>30	21 (87.5)	3 (12.5)			
HER2 score			.23		
0	38 (45.2)	46 (54.8)			
1	3 (27.2)	8 (72.8)			
2	2 (28.5)	5 (71.5)			
cT stage			.019	0.4 (0.10–2.11)	.32
T1	7 (63.6)	4 (36.3)			
T2	34 (44.7)	42 (55.3)			
T3	0 (0)	11 (100)			
T4	2 (50)	2 (50)			
Ki-67, median, IQR	70 (60–80)	60 (50–80)	.52		
Grade			.87		
Grades 1 and 2	8 (40.0)	12 (60.0)			
Grade 3	34 (41.9)	47 (58.1)			
Menopausal status			.20		
Pre-perimenopause	26 (47.2)	29 (52.8)			
Postmenopause	16 (34.7)	30 (65.3)			

cT stage = clinical T stage, HER2 = human epidermal growth factor 2, OR = odds ratio, pCR = pathological complete response, TILs: tumor infiltrating lymphocytes.

### 3.2. TILs and HER2 relationship

The relationship between the TILs and HER2 score was evaluated. Sixteen patients had a TILs score of 0. Of these, 14 (87%) patients had a HER2 score of 0, 1 (6.5%) patient had a HER2 score of 1, and 1 (6.5%) patient had a HER2 score of 2. Sixty-two patients had TILs scores between 1 and 30. Forty-nine (79%) patients had a HER2 score of 0, 9 (14%) patients had a HER2 score of 1, and 4 (7%) patients had a HER2 score of 2. Twenty-four patients had a TIL score > 30. Twenty-one (87%) patients had a HER2 score of 0, 1 patient had a HER2 score of 1 (4.3%), and 2 patients had a HER2 score of 2 (8.7%). In Spearman correlation analysis, no significant relationship was found between HER2 and the TILs score (*P* = .26, CC: 0.14). This is shown in Table 3.

**Table 3:** Relationship between TILs score and HER2 score.

	HER2 score-0	HER2 score-1	HER2 score-2	*P* ^k^
TILs 0	14 (87.6)	1 (6.2)	1 (6.2)	.26
TILs 1–30	49 (79.0)	9 (14.5)	4 (6.5)	
TILs >30	21 (87.5)	1 (4.2)	2 (8.3)	

HER2 = human epidermal growth factor 2, TILs = tumor infiltrating lymphocytes.

^c^Spearman correlation analysis.

### 3.3. Survival outcomes

**Figure 2.**
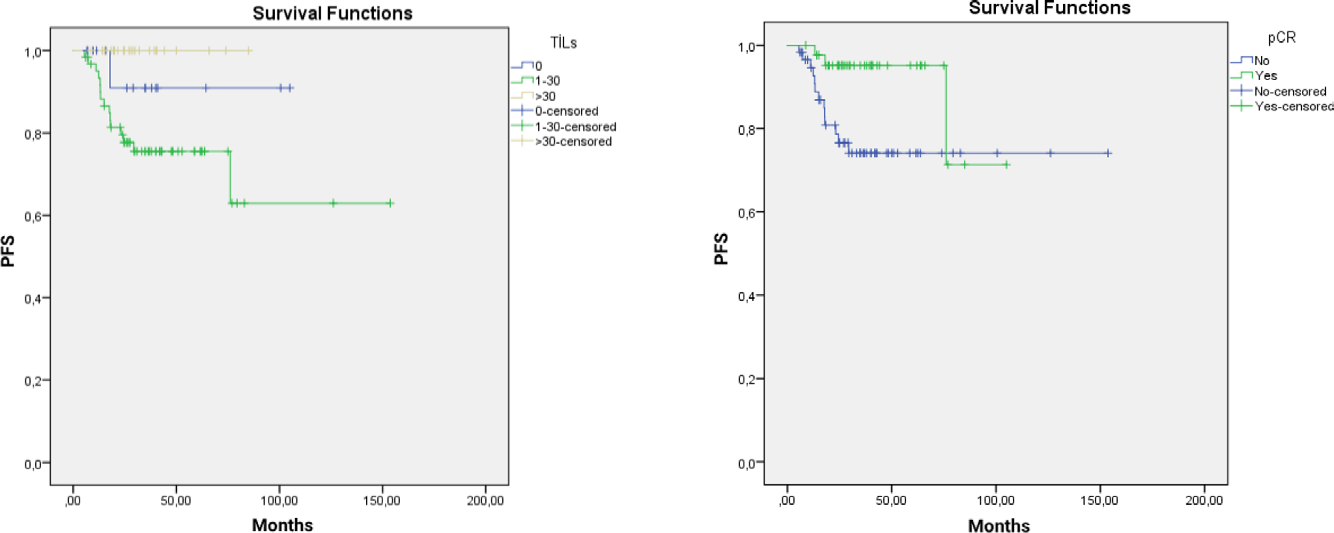
Kaplan–Meier curve according to TILs (A) and pCR (B). pCR = pathological complete response, TILs = tumor infiltrating lymphocytes.

The relationship between TILs score and PFS was evaluated. Because the follow-up period was short, the median PFS could not be reached. In the univariate analysis, a significant association was found between the TILs score and PFS (*P* = .026). PFS was significantly longer in patients with TILs > 30%. A significant relationship was observed between pCR and PFS (*P* = .027). Kaplan–Meier curves are shown in Figure 2A and B. In multivariate analysis, the association between pCR (HR = 0.2, 95% CI = 0.8–1.02, *P* = .054) and PFS approached the threshold of significance but did not reach significance. No significant relationship was found between TILs score and PFS (HR = 0.5, 95% CI = 0.27–1.92, *P* = .73).

## 4. Discussion

In this study, we aimed to evaluate the relationship between TILs and HER2 scores and the relationship of each with pCR in TNBC patients receiving neoadjuvant chemotherapy. We also investigated other variables that could affect pCR. We found that the pCR increased significantly in patients with TILs > 30%. Since the number of patients with TILs 0 was relatively low, although the relationship between the groups with TILs 0 and TILs 1 to 30 did not reach a statistically significant level, there was a significant difference in the group with TILs > 30%. We did not detect a significant relationship between the HER2 score and pCR response. Studies investigating the prognostic and predictive effectiveness of TILs score in TNBC patients have become widespread in recent years. In a study that performed a pooled analysis of 9 studies, the data of 2148 early-stage breast cancer patients were evaluated. Every 10% increase in TILs score corresponds to a 13% risk reduction in invasive disease-free survival and a 17% risk reduction in distant metastasis-free survival.^[11]^ In a pooled analysis of 6 studies evaluating patients with breast cancer receiving neoadjuvant chemotherapy, a 10% increase in the TILs score in the TNBC subgroup was associated with longer disease-free survival. In the same study, increased TILs score was also associated with longer overall survival in TNBC, but no association was found in HER2-positive breast cancer.^[12]^ In all of these studies, patients received only chemotherapy in neoadjuvant therapy. Additionally, the relationship between TILs score and immunotherapy response has also been investigated.^[13]^ In the KEYNOTE-173 study, pathological response rates were determined to increase as both the PD-L1 CPS and the the TILs score increased.^[14]^ All these studies have shown that the TILs score may be a good predictor of treatment response to both chemotherapy and immunotherapy.

The relationship between HER2 score and treatment responses and survival in both hormone receptor-positive breast cancer and TNBC has been investigated in recent years. A study conducted in the Far East, compared HER2-zero TNBC and HER2-low TNBC, revealing that HER2-zero TNBC exhibited a potentially more active immune microenvironment than HER2-low TNBC^[15]^ In light of these data, we examined the relationship between HER2 score and TILs score in this study. No significant relationship was found between HER2 score and TILs score in this study. Our patient population included only TNBC patients who received neoadjuvant chemotherapy, and the number of patients was slightly lower. There is a need for large-scale research on this subject. Additionally, we should not forget that a clear conclusion about the tumor microenvironment cannot be reached with TILs evaluation alone. Therefore, better predictive markers must be identified.

When we evaluated other factors affecting pCR, no significant relationship was found between neodjuvant treatment response and menopausal status. Although studies on TNBC have shown that premenopause is associated with lower PFS,^[16]^ the relationship between menopausal status and neoadjuvant treatment response is not clear. Similarly, no relationship was found between the grade, Ki-67 and pCR.

It has been shown that pCR obtained with neoadjuvant treatment for TNBC is associated with longer PFS and OS.^[17]^ In our study, median PFS could not be reached due to the short follow-up period. Although the median PFS was longer in the pCR group, it did not reach statistical significance. However, we believe that it would not be appropriate to comment on these results with such a short follow-up period.

The main limitations of this study include its retrospective design and the inclusion of a small number of patients. Additionally, due to the retrospective nature of the study, there may be potential patient selection bias. The follow-up period was very short, making it impossible to correctly interpret the PFS information, and our patients could not receive immunotherapy in the neoadjuvant period due to reimbursement conditions in our country. We believe that treatment responses will change with the addition of checkpoint inhibitors to chemotherapy. Considering these shortcomings, we believe that our study is valuable because it examines the variables affecting pCR in a specific group.

## 5. Conclusion

In summary; the TILs score is a good prognostic factor for predicting neoadjuvant chemotherapy response in patients with TNBC. Although we could not find a relationship between HER2 score and pathological response, we belive that this relationship should be investigated in larger scale studies. In this study, we showed that there was no significant relationship between TILs score and HER2 score. Larger molecular studies are required to examine this relationship. A marker that predicts neoadjuvant treatment has not yet been clearly determined. Although TILs and PD-L1 scores have been shown to be related to treatment response, stronger predictive markers are needed.

## Acknowledgments

Thanks to all the staff of University of Marmara Pathology Department and Dr Abdurrahman Yurtaslan Oncology Education and Research Hospital Pathology Department.

## Author contributions

**Conceptualization:** Didem Kurukafa, Yeşim Ağyol, Elif Sertesen Çamöz, Alper Türkel, Sibel Yenidünya, Öztürk Ateş.

**Data curation:** Yeşim Ağyol, Hatice Gülgün Firat, Berkan Karabuğa, Alper Türkel.

**Formal analysis:** Hatice Gülgün Firat, Öztürk Ateş.

**Funding acquisition:** İlknur Deliktaş Onur.

**Investigation:** İlknur Deliktaş Onur, Öztürk Ateş.

**Methodology:** Yeşim Ağyol, İbrahim Vedat Bayoğlu, Öztürk Ateş.

**Project administration:** Elif Sertesen Çamöz, Sibel Yenidünya.

**Resources:** Didem Kurukafa, Berkan Karabuğa, Sibel Yenidünya, Öztürk Ateş.

**Supervision:** İlknur Deliktaş Onur, Elif Sertesen Çamöz, Öztürk Ateş.

**Validation:** İlknur Deliktaş Onur, Hatice Gülgün Firat, Alper Türkel.

**Visualization:** İlknur Deliktaş Onur, Didem Kurukafa.

**Writing – original draft:** İlknur Deliktaş Onur.

**Writing – review & editing:** İlknur Deliktaş Onur, Hatice Gülgün Firat, Öztürk Ateş.
